# Meta-Analysis of Risk Factors for Bile Leakage After Hepatectomy Without Biliary Reconstruction

**DOI:** 10.3389/fsurg.2021.764211

**Published:** 2021-11-01

**Authors:** Ling Tan, Fei Liu, Zi-lin Liu, Jiang-wei Xiao

**Affiliations:** Department of Gastrointestinal Surgery, Clinical Medical College and the First Affiliated Hospital of Chengdu Medical College, Chengdu, China

**Keywords:** surgery, hepatectomy, bile leakage, risk factors, meta-analysis

## Abstract

**Background and Aim:** The risk factors for bile leakage after hepatectomy without biliary reconstruction are controversial. This study investigated the risk factors for bile leakage after hepatectomy without biliary reconstruction.

**Methods:** We searched databases (Embase (Ovid), Medline (Ovid), PubMed, Cochrane Library, and Web of Science) for articles published between January 1, 2000, and May 1, 2021, to evaluate the risk factors for bile leakage after hepatectomy without biliary reconstruction.

**Results:** A total of 16 articles were included in this study, and the overall results showed that sex (OR: 1.21, 95% CI: 1.04–1.42), diabetes (OR: 1.21, 95% CI: 1.05–1.38), left trisectionectomy (OR: 3.53, 95% CI: 2.32–5.36), central hepatectomy (OR: 3.28, 95% CI: 2.63–4.08), extended hemihepatectomy (OR: 2.56, 95% CI: 1.55–4.22), segment I hepatectomy (OR: 2.56, 95% CI: 1.50–4.40), intraoperative blood transfusion (OR:2.40 95%CI:1.79–3.22), anatomical hepatectomy (OR: 1.70, 95% CI: 1.19–2.44) and intraoperative bleeding ≥1,000 ml (OR: 2.46, 95% CI: 2.12–2.85) were risk factors for biliary leakage. Age >75 years, cirrhosis, underlying liver disease, left hepatectomy, right hepatectomy, benign disease, Child–Pugh class A/B, and pre-operative albumin <3.5 g/dL were not risk factors for bile leakage after hepatectomy without biliary reconstruction.

**Conclusion:** Comprehensive research in the literature revealed that sex, diabetes, left trisectionectomy, central hepatectomy, extended hemihepatectomy, segment I hepatectomy, intraoperative blood transfusion, anatomical hepatectomy and intraoperative bleeding ≥1,000 ml were risk factors for biliary leakage.

## Introduction

With deepening of the understanding of liver diseases and the development of hepatectomy techniques, the indications for liver resection have been continuously expanded, and the incidence of perioperative complications and mortality have been significantly reduced, but the incidence of bile leakage has not changed significantly (3.1 ~ 28.0%) (1). Miura et al. reported in 2016 that the biliary leakage rate of 14,970 patients who underwent more than segment I hepatectomy recorded by the Japanese National Clinical Database from 2011 to 2012 was 8.0% (2). Yamashita et al. reported in 2020 that the bile leakage rate of 10,102 patients who underwent complex hepatectomy from 2015 to 2017 was 7.2% (3). These findings show that with the development of technology, the incidence of bile leakage after hepatectomy has not been significantly reduced, and bile leakage is still a difficult clinical problem.

Bile leakage can cause severe complications such as post-operative abdominal infection and sepsis, prolong hospitalization, increase treatment costs, and even cause death (4). Studies have shown that bile leakage may inhibit liver regeneration and promote bile duct malignancies (5), thus affecting the prognosis of patients. However, the lack of standardization for the treatment of biliary leakage often delays the optimal treatment window, aggravates the patient's condition, and causes serious trauma to the patient. Clarifying the risk factors for biliary leakage, avoiding and preventing related risk factors, and minimizing the incidence of biliary leakage are particularly important after hepatectomy.

We collected relevant research reports on the risk factors for biliary leakage and further clarified the related risk factors for biliary leakage after hepatectomy without biliary reconstruction by means of meta-analysis, aiming to provide a reference for the clinical prevention and treatment of biliary leakage.

## Materials and Methods

For this systematic review, we adhered to the Meta-analysis of Observational Studies guidelines and the Reporting Items for Systematic reviews and Meta-Analysis (PRISMA) statement (6).

### Search Strategy and Inclusion Criteria

A systematic search was performed based on the following databases: PubMed, Embase (Ovid), Medline (Ovid), Cochrane Library and Web of Science from January 1, 2000, to May 1, 2021. We used ‘hepatectomy,’ ‘liver resection,’ ‘bile leakage,’ ‘biliary fistula,’ ‘risk factor,’ and corresponding free words to search the literature in the above databases, with the language restricted to English. Literature inclusion standard: 1. literature studied the influence of different factors in the perioperative period on the occurrence of bile leakage after hepatectomy; 2. the sample size is at least 100 cases. Literature inclusion standard: 1. studies involving biliary reconstruction; 2. the sample size is less than 100 cases;3. the definition of bile leakage does not meet the ISGLS standard.

### Bile Leakage Risk Factor Outcomes of Interest

The outcomes of interest included: age >75 years, sex, pre-operative albumin <3.5 g/dL, Child–Pugh class A/B, underlying liver disease, liver cirrhosis, diabetes, benign disease, intraoperative bleeding≥1,000 mL, intraoperative blood transfusion, segment I hepatectomy, left trisectionectomy, extended hemihepatectomy, central hepatectomy, left hepatectomy, right hepatectomy and anatomical hepatectomy.

### Definition of Bile Leakage

This study used the International Study Group of Liver Surgery to define bile leakage (7), that is, the presence of bilirubin in the abdominal drainage or intraperitoneal fluid on or after the third day following surgery or the need for intervention due to bile collection or biliary peritonitis.

### Data Extraction and Quality Assessment

First, TL and LF reviewed the titles and abstracts of all identified studies. Next, the same two reviewers independently reviewed the full texts of potentially eligible studies. If any disagreements arose, a third reviewer (LZL) was consulted, and a discussion ensued until a consensus was reached. All the data were independently extracted by TL and LF and compared for consistency. The following relevant information was extracted from all the included literature: first author, year of publication, country, journal, the number of patients, age, and surgery. The quality of the included studies was assessed using the Newcastle Ottawa Scale (NOS), with a maximum of nine points per study. Studies with a score <5 were considered low-quality studies and excluded. Publication bias was assessed by visual inspection of the symmetry of a funnel plot.

### Statistical Analysis

We used the R (version 4.1.0) Meta package for meta-analysis. We calculated the odds ratios (ORs) and 95% confidence intervals (CIs) of different factors in the biliary leakage group and the non-bile leakage group after hepatectomy and the ORs and 95% CIs of multiple studies combined. The *I*^2^ statistic was used to assess heterogeneity; *I*^2^ > 50% was considered indicative of heterogeneity, and the random effects model is adopted, otherwise, the fixed effects model is adopted.

## Results

**Figure 1:**
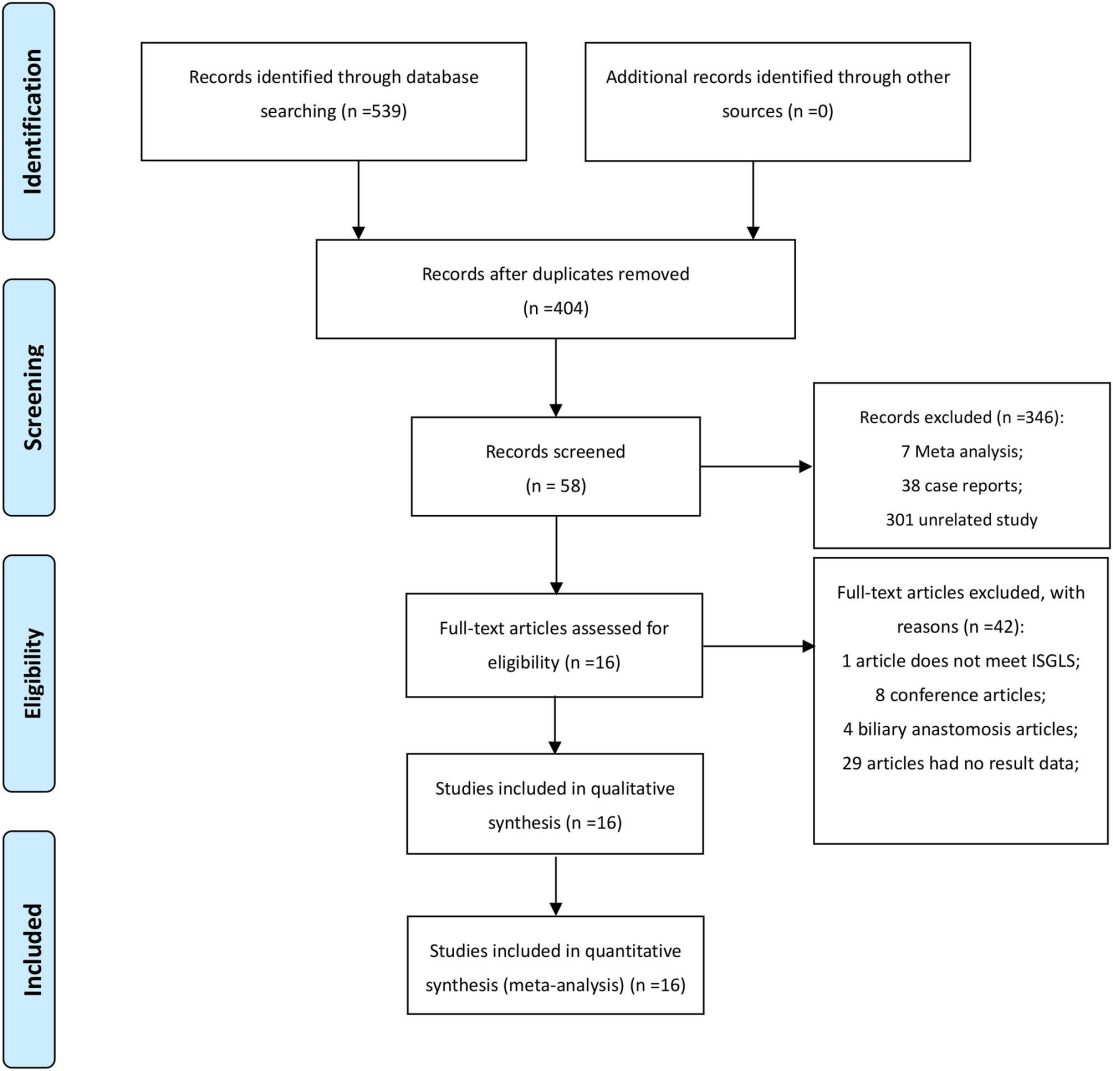
PRISMA flow chart of search strategy and research selection.

**Figure 2:**
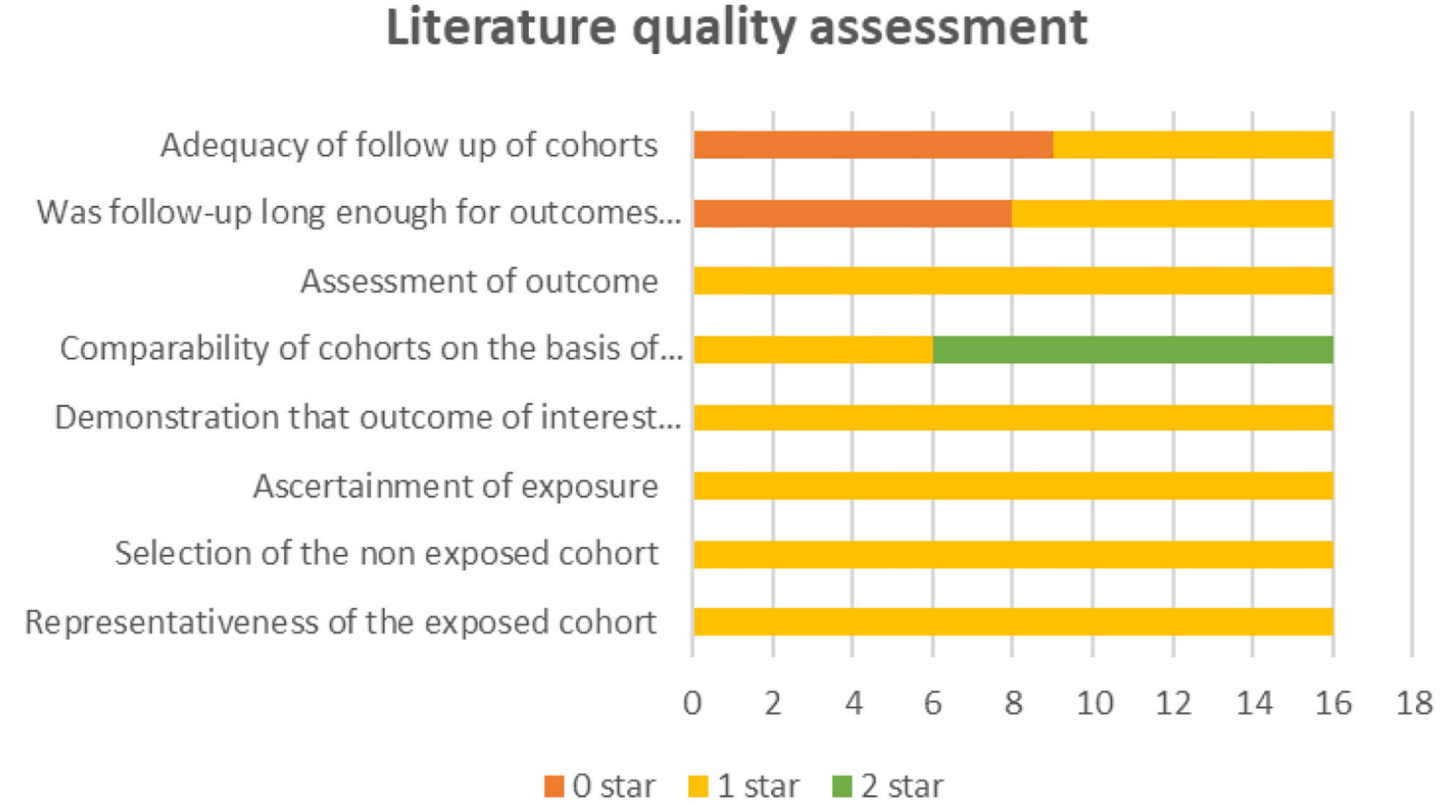
Literature quality assessment based on the Newcastle-Ottawa Scale.

After removing duplicates, we obtained 404 publications from PubMed, Medline (Ovid), Embase (Ovid), Web of Science and Cochrane Library (Figure 1). A total of 16 publications (3, 8–22) and 16,051 hepatectomy patients were eligible for inclusion. Table 1 shows the characteristics of the retrieved publications. Among the patients, 1,274 had biliary leakage, and the incidence of biliary leakage was 7.9%. The NOS scores of the nine studies ranged from 6 to 8 (Figure 2). The literature collected was considered qualified.

**Table 1:** Characteristics of studies included in meta-analysis.

**Author**	**Year**	**Journal**	**Country**	***N***	**Bile leakage rate**	**Age**	**Operation method**
Yamashita, YI	2020	J Hepatobiliary Pancreat Sci	Japan	10,102	7.2%	–	Laparotomy
Sakamoto, K	2016	World J Surg	Japan	334	9.0 %	68 (32–87)	–
Sadamori, H	2013	Br J Surg	Japan	359	12.8%	–	Laparotomy
Sadamori, H	2010	J Hepatobiliary Pancreat Sci	Japan	293	12.9%	–	Laparotomy
Panaro, F	2016	Hepatobiliary Pancreat Dis Int	France	411	10.2%	–	–
Nakano, R	2018	Int J Surg	Japan	556	5.0%	69.8 ± 9.1 vs 69.9 ± 12.4	–
Nagano, Y	2003	World J Surg	Japan	313	5.4%	70.1 vs. 61.7	–
Capussotti, L	2006	Arch Surg	Italy	610	3.6%	61.65 (2–86) vs. 63.18 (49–78)	–
Cauchy, F	2016	Surg Endosc	France	223	13.5%	63.8 (24.1–86.2) vs. 62.5 (23.9–84.0)	Laparoscopic
Donadon, M	2016	World J Surg	Italy	475	8.0%	66 (23–85)	–
Erdogan, D	2008	Dig Surg	The Netherlands	234	6.8%	55.1 ± 1.0 vs. 59.2 ± 3.0	–
Guillaud, A	2013	HPB	France	1001	8.0%	64 (16–90)	Laparotomy and laparoscopic
Harimoto, N	2020	Surg Today	Japan	270	4.4%	68 (28–89)	Laparotomy
Haruki, K	2013	Langenbecks Arch Surg	Japan	105	8.6%	65.0 ± 10.0	–
Ishii, H	2011	Dig Surg	Japan	247	10.5%	63 (21–85) vs. 62 (22–81)	–
Kajiwara, T	2016	BMC Surg	Japan	518	15.6%	68 (20–84) vs 68 (44–84)	–

### Bile Leakage Risk Factor Outcomes of Interest

#### Sex

**Figure 3:**
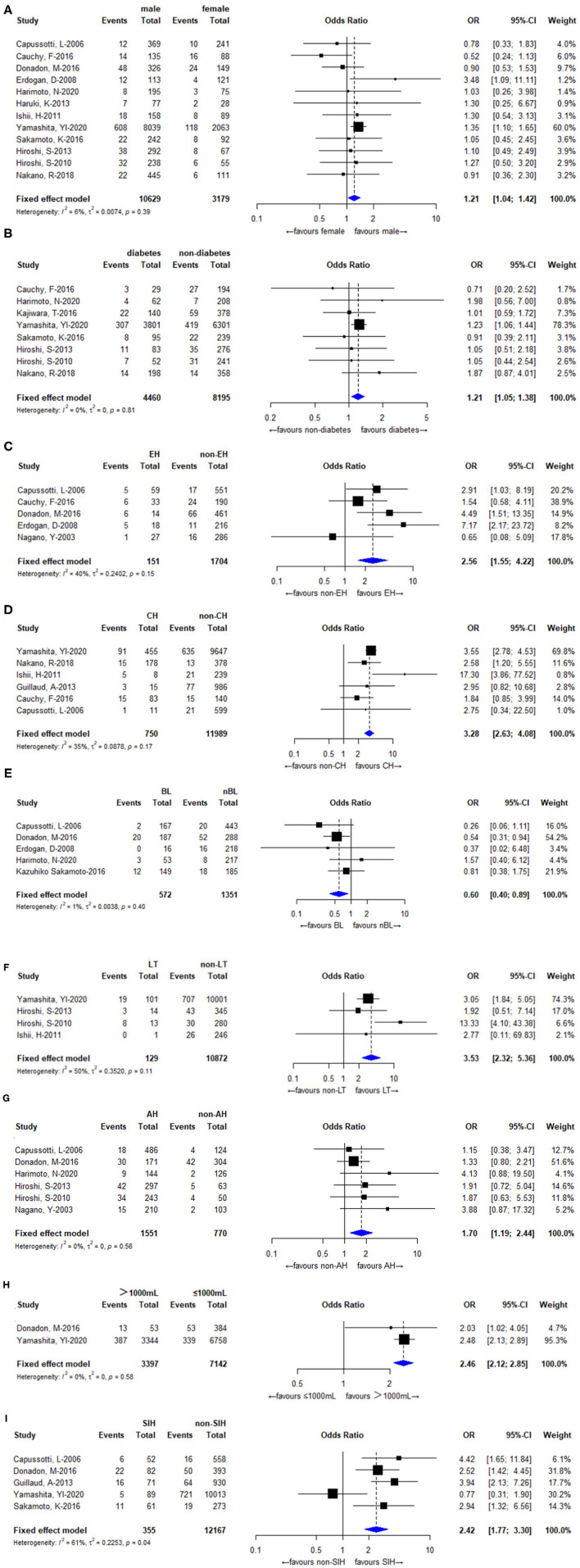
Forest plots for the meta-analyses. **(A)** Sex; **(B)** Diabetes; **(C)** Extended hemihepatectomy (EH); **(D)** Central hepatectomy (CH); **(E)** Liver cirrhosis (LC); **(F)** Left trisectionectomy (LT); **(G)** Anatomical hepatectomy (AH); **(H)** Bleeding; **(I)** Segment I hepatectomy (SIH).

Twelve (3, 8–11, 13–15, 18, 20–22) of the 16 included studies reported the influence of sex on the occurrence of bile leakage after hepatectomy. The overall outcomes showed that males had an increased incidence of bile leakage after hepatectomy (OR: 1.21, 95% CI: 1.04–1.42; *I*^2^ = 6% *P* = 0.39), as shown in Figure 3A.

#### Diabetes

Eight (3, 9, 13, 16, 18, 20–22) of the 16 included studies reported the influence of diabetes on the occurrence of bile leakage after hepatectomy. The overall outcomes showed that diabetes increased the incidence of bile leakage after hepatectomy (OR: 1.21, 95% CI: 1.05–1.38, *I*^2^ = 0% *P* =0.81), as illustrated in Figure 3B.

#### Extended Hemihepatectomy

Five (8–11, 17) of the 16 included studies reported the influence of extended hemihepatectomy on the occurrence of bile leakage after hepatectomy. The overall outcomes showed that extended hemihepatectomy increased the incidence of bile leakage after hepatectomy (OR: 2.56, 95% CI: 1.55–4.22, *I*^2^ = 40% *P* = 0.15), as illustrated in Figure 3C.

#### Central Hepatectomy

Six (3, 8, 9, 12, 15, 18) of the 16 included studies reported the influence of central hepatectomy on the occurrence of bile leakage after hepatectomy. The overall outcomes showed that central hepatectomy increased the incidence of bile leakage after hepatectomy (OR: 3.28, 95% CI: 2.63–4.08), *I*^2^ = 35% *P* =0.17), as shown in Figure 3D.

#### Liver Cirrhosis

Five (8, 10, 11, 13, 22) of the 16 included studies reported the influence of liver cirrhosis on the occurrence of bile leakage after hepatectomy. The results showed that liver cirrhosis reduced the incidence of bile leakage after hepatectomy (OR: 0.60, 95% CI: 0.40–0.89, *I*^2^ = 1% *P* = 0.40), as shown in Figure 3E.

#### Left Trisectionectomy

Four (3, 15, 20, 21) of the 16 included studies reported the influence of left trisectionectomy on the occurrence of bile leakage after hepatectomy. The overall outcomes showed that left trisectionectomy increased the incidence of bile leakage after hepatectomy (OR: 3.53, 95% CI: 2.32–5.36), *I*^2^ = 50% *P* = 0.11), as shown in Figure 3F.

#### Anatomical Hepatectomy

Six (8, 10, 13, 17, 20, 21) of the 16 included studies reported the influence of anatomical hepatectomy on the occurrence of bile leakage after hepatectomy. The overall outcomes showed that anatomical hepatectomy did not affect the occurrence of bile leakage after hepatectomy (OR: 1.70, 95% CI: 1.19–2.44, *I*^2^ = 0% *P* = 0.56), as shown in Figure 3G.

#### Intraoperative Bleeding ≥1,000 mL

Two (3, 10) of the 16 included studies reported the influence of bleeding ≥1,000 ml on the occurrence of bile leakage after hepatectomy. The overall outcomes showed that bleeding ≥1,000 ml increased the incidence of bile leakage after hepatectomy (OR: 2.46, 95% CI: 2.12–2.85), *I*^2^ = 0% *P* = 0.58), as shown in Figure 3H.

#### Segment I Hepatectomy

Five (3, 8, 9, 12, 22) of the 16 included studies reported the influence of segment I hepatectomy on the occurrence of bile leakage after hepatectomy. The overall outcomes showed that segment I hepatectomy increased the incidence of bile leakage after hepatectomy (OR: 2.56, 95% CI: 1.50–4.40, *I*^2^ = 61% *P* = 0.04), as shown in Figure 3I.

#### Age>75 Years

Four (3, 8, 9, 12) of the 16 included studies reported the influence of age >75 years on the occurrence of bile leakage after hepatectomy. The overall outcomes showed that age >75 years did not affect the occurrence of bile leakage after hepatectomy (OR: 1.12, 95% CI: 0.97–1.31, *I*^2^ = 32% *P* = 0.22), as shown in Supplementary Figure 1A.

#### Underlying Liver Disease

Nine (8, 10, 11, 13, 15, 16, 18, 20, 21) of the 16 included studies reported the influence of underlying liver disease on the occurrence of bile leakage after hepatectomy. The overall outcomes showed that underlying liver disease did not affect the occurrence of bile leakage after hepatectomy (OR: 0.91, 95% CI: 0.70–1.19, *I*^2^ = 0% *P* = 0.62), as shown in Supplementary Figure 1B.

#### Left Hepatectomy

Six (3, 8, 11, 15, 18, 22) of the 16 included studies reported the influence of left hepatectomy on the occurrence of bile leakage after hepatectomy. The overall outcomes showed that left hepatectomy did not affect the occurrence of bile leakage after hepatectomy (OR: 0.90, 95% CI: 0.72–1.13, *I*^2^ = 0% *P* = 0.62), as shown in Supplementary Figure 1C.

#### Right Hepatectomy

Five (8, 9, 15, 18, 22) of the 16 included studies reported the influence of right hepatectomy on the occurrence of bile leakage after hepatectomy. The overall outcomes showed that right hepatectomy did not affect the occurrence of bile leakage after hepatectomy (OR: 0.90, 95% CI: 0.72–1.13, *I*^2^ = 0% *P* = 0.62), as shown in Supplementary Figure 1D.

#### Benign Disease

Three (8, 9, 11) of the 16 included studies reported the influence of benign disease on the occurrence of bile leakage after hepatectomy. The overall outcomes showed that benign disease did not affect the occurrence of bile leakage after hepatectomy (OR: 0.52, 95% CI: 0.22–1.26, *I*^2^ = 0% *P* = 1.00), as shown in Supplementary Figure 1E.

#### Child-Pugh Class A/B

Six (13, 15, 16, 18, 20, 21) of the 16 included studies reported the influence of Child–Pugh class on the occurrence of bile leakage after hepatectomy. The overall outcomes showed that Child–Pugh class did not affect the occurrence of bile leakage after hepatectomy (OR: 0.68, 95% CI: 0.39–1.19, *I*^2^ = 27% *P* = 0.23), as shown in Supplementary Figure 1F.

#### Intraoperative Blood Transfusion

Eight (9–14, 20, 21) of the 16 included studies reported the influence of intraoperative blood transfusion on the occurrence of bile leakage after hepatectomy. The overall outcomes showed that blood transfusion did not affect the occurrence of bile leakage after hepatectomy (OR: 1.57, 95% CI: 0.75–3.30, *I*^2^ = 84% *P* <0.01), as shown in Supplementary Figure 1G.

#### Pre-operative Albumin <3.5 g/dL

Three (3, 20, 21) of the 16 included studies reported the influence of pre-operative albumin on the occurrence of bile leakage after hepatectomy. The overall outcomes showed that pre-operative albumin <3.5 g/dL did not affect the occurrence of bile leakage after hepatectomy [OR: 0.91 (95% CI: 0.48–1.75), *I*^2^ = 68% *P* = 0.04], as shown in Supplementary Figure 1H.

### Sensitivity Analysis

We conducted a sensitivity analysis on heterogeneity factors (segment I hepatectomy, blood transfusion, pre-operative albumin <3.5 g/dL) and found the source of heterogeneity (as shown in Supplementary Figure 2). The analysis revealed the following results (as shown in Supplementary Figure 3): the segment I hepatectomy OR was 3.13 (2.20–4.44), and heterogeneity tests showed that the trials did not have heterogeneity (*I*^2^ = 0%; *P* = 0.66); the intraoperative blood transfusion OR was 2.40 (1.79–3.22), and heterogeneity tests showed that the trials did not have heterogeneity (*I*^2^ = 0%; *P* = 0.57); the pre-operative albumin <3.5 g/dL OR was 0.62 (0.34–1.14), and heterogeneity tests showed that the trials did not have heterogeneity (*I*^2^ = 0%; *P* = 0.80). After heterogeneity was excluded, the results for segment 1 hepatectomy and pre-operative chemotherapy were consistent with the results without heterogeneity exclusion. After excluding heterogeneity, the results showed that intraoperative blood transfusion increased the incidence of bile leakage after hepatectomy.

### Publication Bias

**Figure 4:**
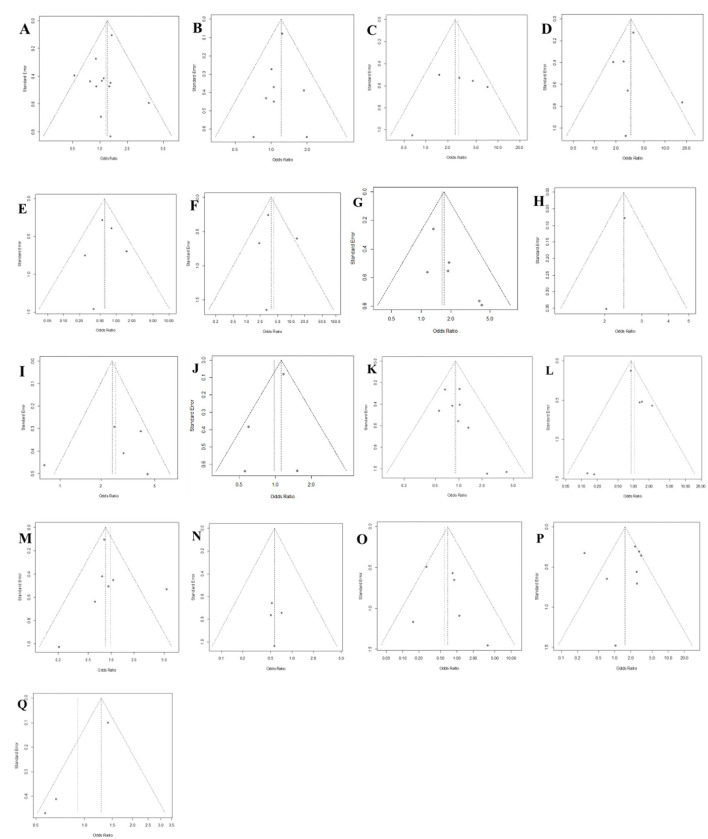
Funnel plot of publication bias in the meta-analysis. **(A)** Sex; **(B)** Diabetes; **(C)** Extended hemihepatectomy; **(D)** Central hepatectomy; **(E)** Liver cirrhosis; **(F)** Left trisectionectomy; **(G)** Anatomical hepatectomy; **(H)** Bleeding; **(I)** Segment I hepatectomy; **(J)** Age; **(K)** Underlying liver disease; **(L)** Left hepatectomy; **(M)** Right hepatectomy; **(N)** Benign disease; **(O)** Child-Pugh class A/B; **(P)** Intraoperative blood transfusion; **(Q)** Pre-operative albumin <3.5 g/dL.

Publication bias was assessed by visual examination of the symmetry of the funnel plot. Our funnel plot showed no publication bias (Figure 4).

## Discussion

Research on risk factors related to bile leakage after hepatectomy has expanded from discussions of surgical factors to the entire perioperative period. The relevant factors included in the study were patient characteristics, surgical methods and post-operative treatment (3, 8, 23). Nevertheless, the conclusions of various studies are still controversial, and maximally clarifying the related risk factors for bile leakage is very important, which will be helpful for us to prevent and reduce the occurrence of bile leakage.

Our research results show that among the risk factors for bile leakage, risk factors related to the patient are sex and diabetes, while risk factors related to surgery are left trisectionectomy, central hepatectomy, extended hemihepatectomy, segment 1 resection, intraoperative blood transfusion, anatomical hepatectomy and intraoperative bleeding >1,000 ml. However, advanced age (age >75 years), pre-operative albumin <3.5 g/dL, underlying liver disease, benign disease, hemihepatectomy, and Child–Pugh class A/B are not biliary risk factors for leakage.

Previous studies have shown that sex is a risk factor for bile leakage after hepatectomy without biliary reconstruction (3, 11). The results of our research are consistent with those of previous studies. The influence of sex hormones may be pertinent, but no relevant evidence is available at present, and further research is needed to obtain the specific influence mechanism.

Our research results fully show that diabetes is a high-risk factor for bile leakage. Diabetes is also a high-risk factor for perioperative complications. Diabetes increases the risk of post-operative infections, prolongs the hospital stay (24, 25), and even increases the mortality rate during the perioperative period (26). However, the impact of diabetes on liver resection has been controversial, especially the influence of bile leakage (26). Diabetes can cause microcirculation disorders and affect tissue healing and is generally considered an independent risk factor for bile leakage (3, 27). Research by Yamamoto et al. (28) pointed out that diabetes can damage the residual liver after hepatectomy and affect healing of the cut surface tissue, which may increase the risk of post-operative bile leakage. Therefore, reasonable blood glucose control before surgery is essential to prevent post-operative bile leakage.

Although the relationship between the type of hepatectomy and biliary leakage is not clear, previous studies mostly speculated that resection of the central segment of the liver with hilar exposure was a high-risk factor for biliary leakage (15, 17, 23, 29, 30). However, Sadamori et al. believe that the type of hepatectomy is not a risk factor for bile leakage (21). Even in the case of a large section area and exposure of the Glisson system, as long as the pre-operative liver function assessment is reasonable and surgery is meticulously performed, no bile leakage is usually observed after the operation. Our conclusions show that central hepatectomy, segment I resection, and left trisectionectomy are associated with a higher incidence of bile leakage. Due to the anatomical position, during resection of segment 1 and the central liver segment (S4, S5, S8), the main Glisson system around the hilum is easily damaged, thus causing bile leakage. Central hepatectomy involves a larger resection area, and no tissue coverage may also be one of the reasons for post-operative bile leakage (23). In previous studies, left trisectionectomy was also considered a high-risk factor for bile leakage (3, 23, 30), A large tangent area (31) and the right posterior bile duct often merge into the left bile duct, which may cause intraoperative bile duct damage and bile leakage (32). The pumping action of the right diaphragm increases the residual right hepatic bile duct pressure and increases bile leakage (33). Notably, for the more common hemihepatectomy, our results show that neither left hepatectomy nor right hepatectomy is a risk factor for bile leakage, possibly because hemihepatectomy involves less manipulation in the central area of the hepatic hilum. Therefore, resection of the central area during hepatectomy may lead to a corresponding increase in the risk of bile leakage, which must be comprehensively considered.

The choice of resection method for malignant liver tumors has always been a controversial topic. A meta-analysis by Jiao S et al. showed that anatomical hepatectomy is superior to non-anatomical hepatectomy in terms of the long-term survival rate of patients with HCC (34). Rahbari et al. (35) pointed out that anatomical hepatectomy is a risk factor for bile leakage, and given the significant adverse effects of complications after hepatectomy on the long-term prognosis of malignant liver tumors, caution is recommended when considering surgical methods. Anatomical liver resection requires too much manipulation of the Glisson ligaments, and resection of the central area of the hepatic portal region may increase the occurrence of bile leakage. However, to ensure a radical cure and a prognostic effect of the tumor, we must choose a reasonable surgical procedure based on the advantages and disadvantages. Although our results further support this view, unfortunately, we have included limited literature and insufficient evidence, and more studies are needed to further verify this conjecture in the future.

Our research indicates that intraoperative bleeding ≥1,000 ml and intraoperative blood transfusion are risk factors for post-operative bile leakage, possibly due to the combined effects of massive blood loss during hepatectomy, intraoperative hepatic blood flow obstruction, blood transfusion, etc. (36–38), which may cause and aggravate liver ischemia and reperfusion injury, affect liver function recovery, and cause bile leakage. However, the number of included studies was small, and the evidence was obviously insufficient; therefore, this result requires further confirmation.

This research found that sex, diabetes, left trisectionectomy, central hepatectomy, extended hemihepatectomy, segment I hepatectomy, intraoperative blood transfusion, anatomical hepatectomy and intraoperative bleeding ≥1,000 ml were risk factors for biliary leakage. However, this meta-analysis was mainly limited to the inclusion of only retrospective research data; retrospective research tends to introduce bias. In addition, due to the large time span of the included studies, technological development, and differences in surgical instruments, the results of the study may be biased. At the same time, due to the diversity of liver resection methods, the data in the studies are quite different, resulting in a relative lack of analysis of surgical data, which is also an obvious shortcoming of this study. We hope that more high-quality RCT results will be obtained in the future to guide our understanding of the risk factors for bile leakage.

## Conclusion

Comprehensive research in the literature showed that male sex, diabetes, left trisectionectomy, central hepatectomy, extended hemihepatectomy, segment I hepatectomy, intraoperative blood transfusion, anatomical hepatectomy and intraoperative bleeding ≥1,000 ml were risk factors for biliary leakage.

## Data Availability Statement

The original contributions presented in the study are included in the article/Supplementary Material, further inquiries can be directed to the corresponding author/s.

## Author Contributions

LT and FL: acquisition of data, analysis and interpretation of data, drafting the article, and final approval. Z-lL: interpretation of data, revising the article, and final approval. J-wX: conception and design of the study, critical revision, and final approval. All authors contributed to the article and approved the submitted version.

## Funding

This research was supported by the National Natural Science Foundation of China, No. 81070378 and 81270561; and High-level Talents Introduction Fund for the First Affiliated Hospital of Chengdu Medical College, NO. CYFY2018GQ17.

## Conflict of Interest

The authors declare that the research was conducted in the absence of any commercial or financial relationships that could be construed as a potential conflict of interest.

## Publisher's Note

All claims expressed in this article are solely those of the authors and do not necessarily represent those of their affiliated organizations, or those of the publisher, the editors and the reviewers. Any product that may be evaluated in this article, or claim that may be made by its manufacturer, is not guaranteed or endorsed by the publisher.
