# Supplementary material for: Meta-Analysis of Risk Factors for Bile Leakage After Hepatectomy Without Biliary Reconstruction
Source: Front Surg. 2021 Nov 1;8:764211. doi: 10.3389/fsurg.2021.764211 (PMC8591075; doi:10.3389/fsurg.2021.764211)
Supplement: Supplementary file 1 [file Data_Sheet_1.docx]

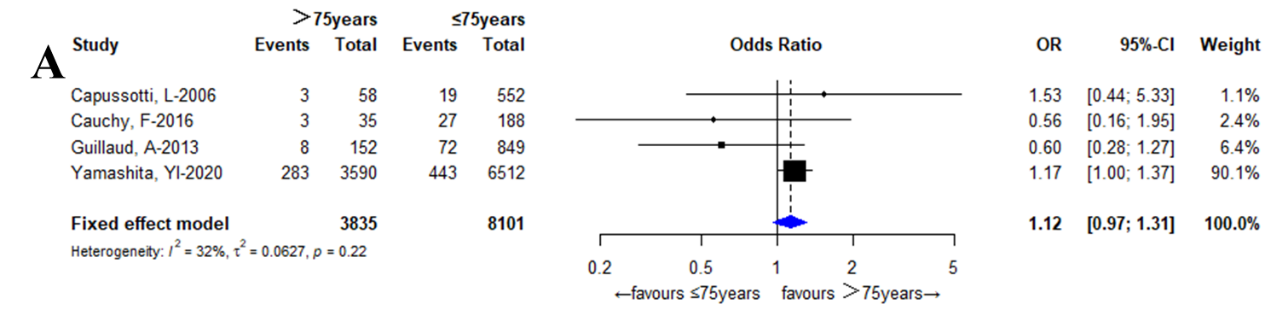


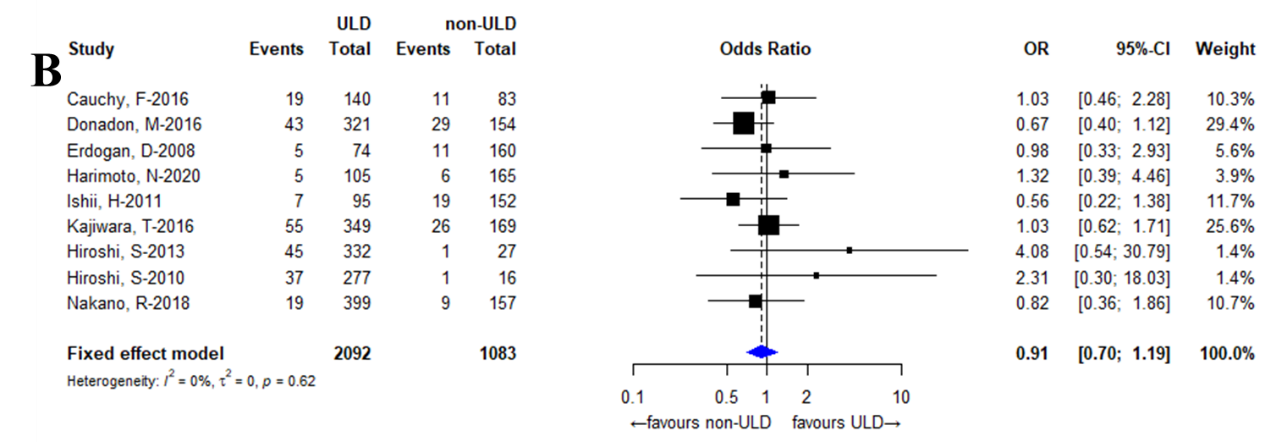


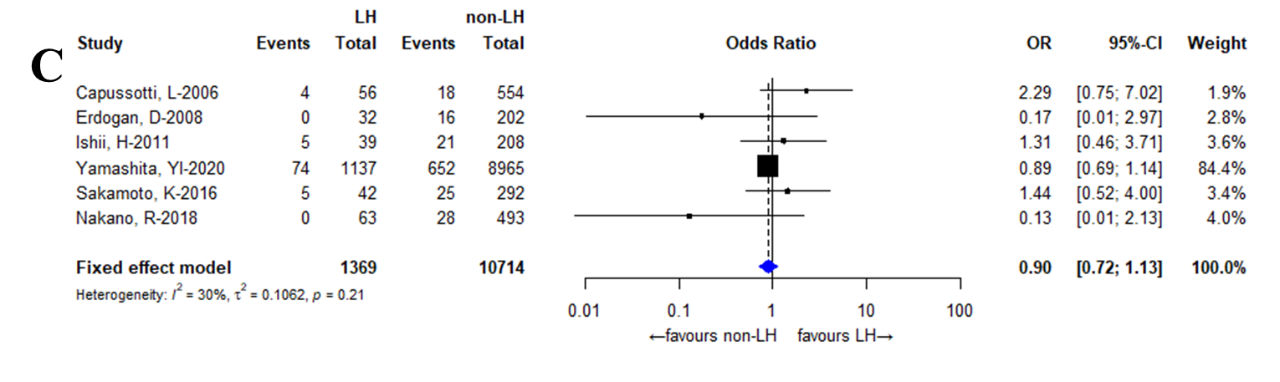


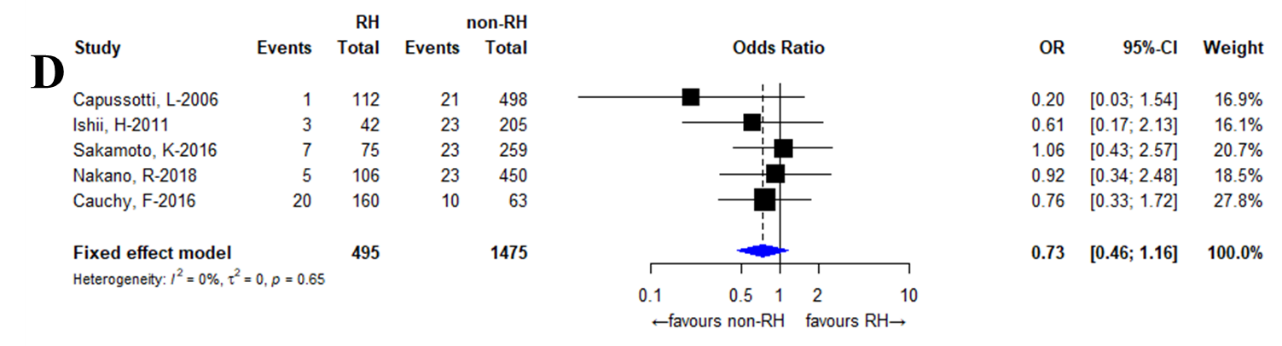


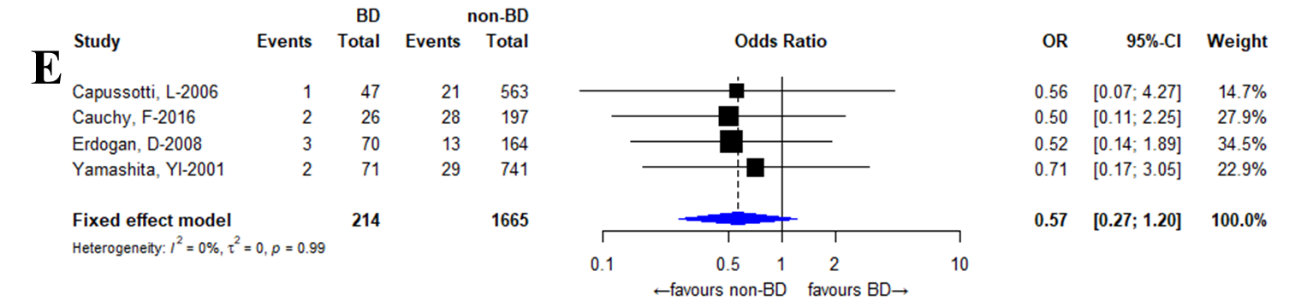


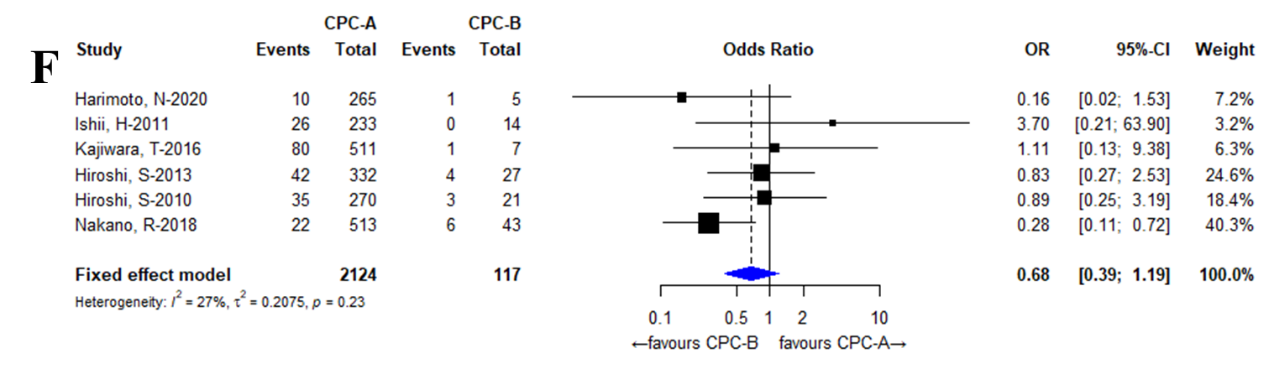


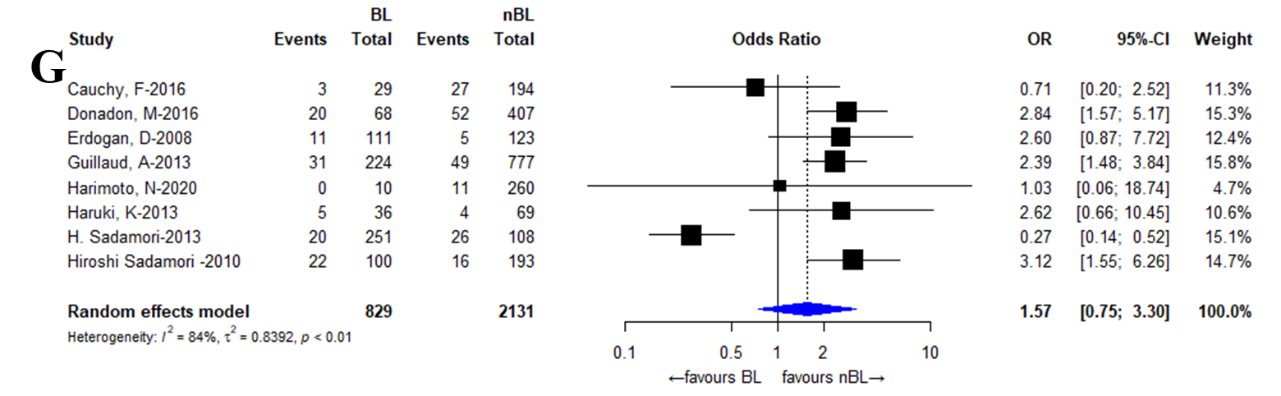


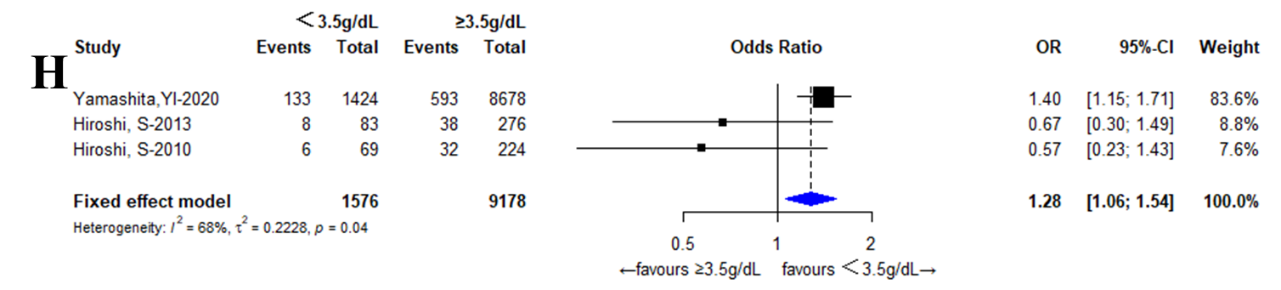


**Supplementary Fig. 1 Forest plots for the meta-analyses. A:** Age; **B:** Underlying liver disease (ULD); **C:** Left hepatectomy (LH); **D:** Right hepatectomy (RH); **E:** Benign disease (BD); **F:** Child-Pugh class A/B (CPC); **G:** Intraoperative blood transfusion (BL); **H:** Preoperative albumin<3.5g/dL.


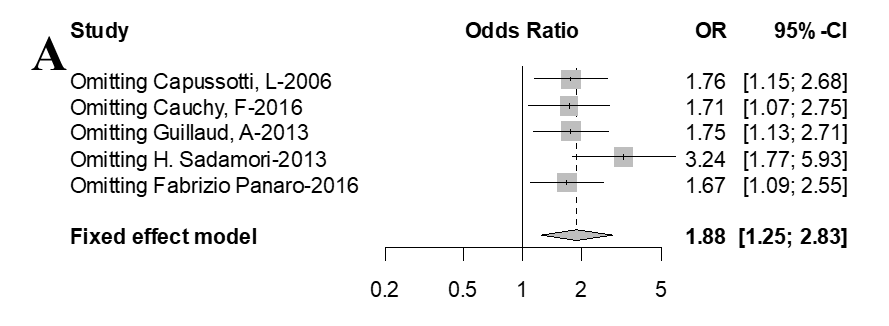

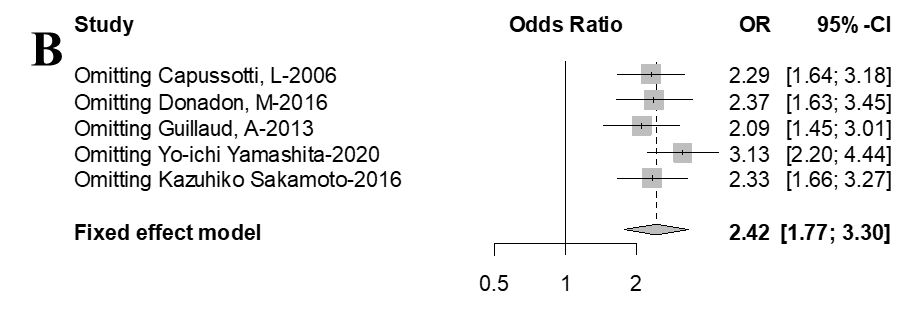


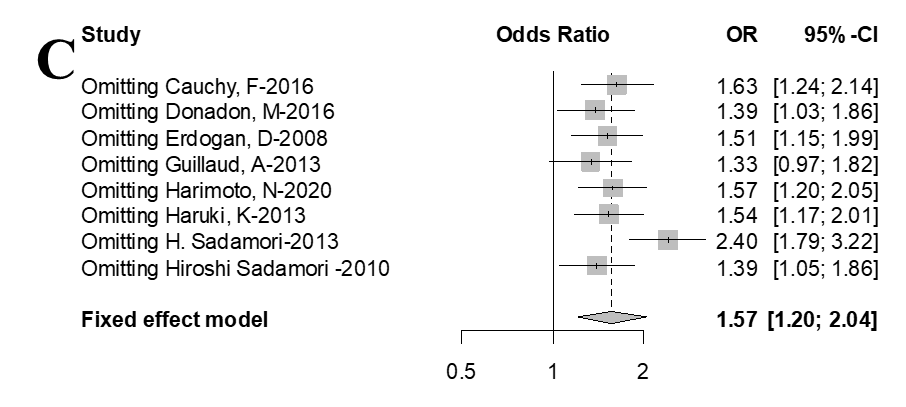


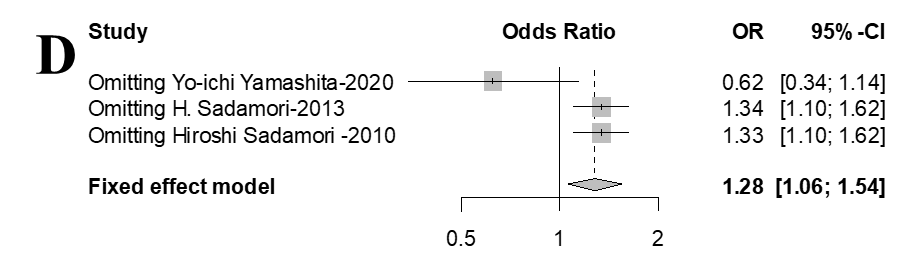


**Supplementary Fig. 2 Sensitivity map for the meta-analysis. A:** Drainage tube; **B:** Segment I hepatectomy; **C:** Intraoperative blood transfusion; **D:** Preoperative albumin<3.5g/dL.


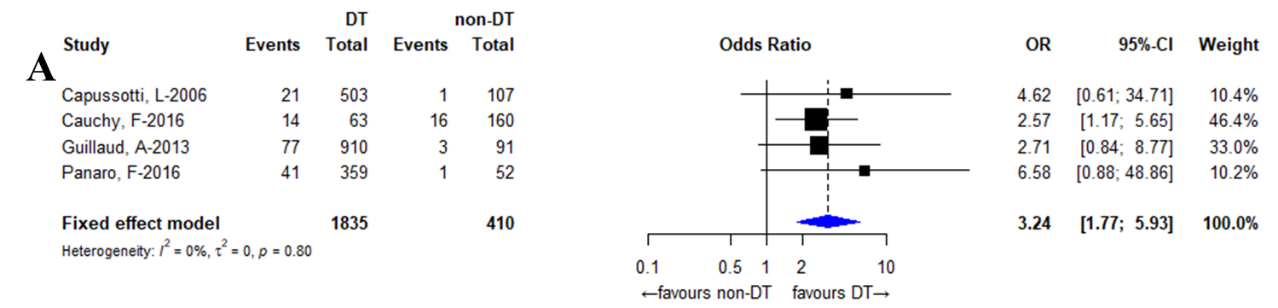


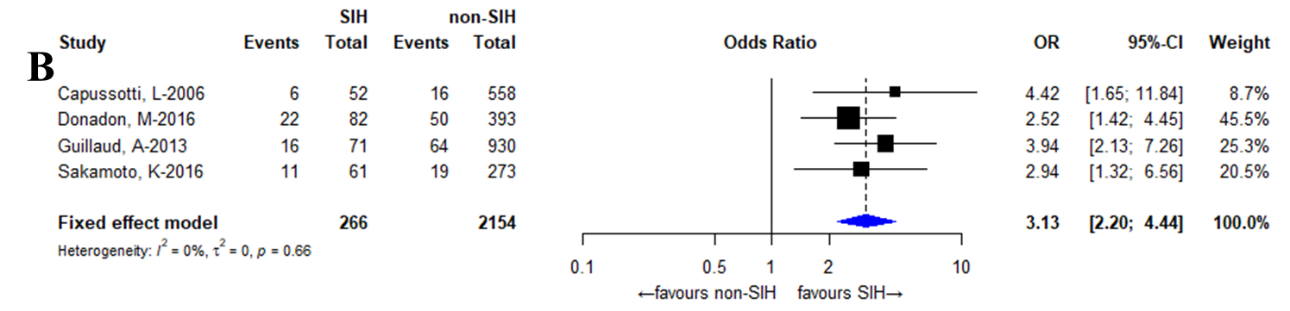


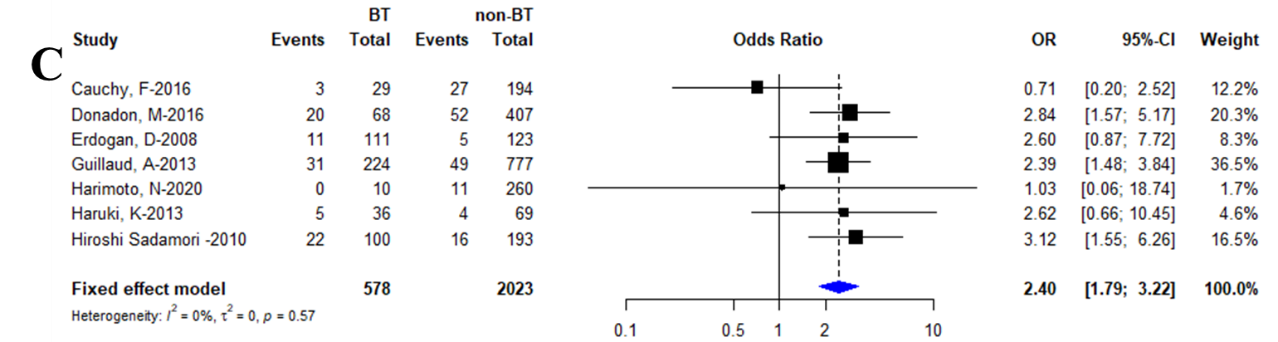


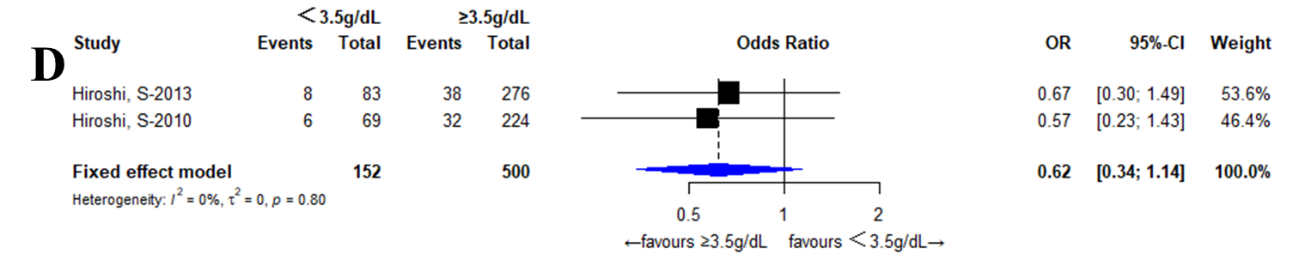


**Supplementary Fig. 3 Forest plot for meta-analysis after excluding heterogeneity. A:** Drainage tube (DT)**; B:** Segment I hepatectomy (SIH)**; C:** Intraoperative blood transfusion (BT)**; D:** Preoperative albumin<3.5g/Dl.
